# Disparities Contributing to Late-Stage Diagnosis of Lung, Colorectal, Breast, and Cervical Cancers: Rural and Urban Poverty in Florida

**DOI:** 10.3390/cancers15215226

**Published:** 2023-10-31

**Authors:** Jaclyn M. Hall, Rahma S. Mkuu, Hee Deok Cho, Jennifer N. Woodard, Frederic J. Kaye, Jiang Bian, Elizabeth A. Shenkman, Yi Guo

**Affiliations:** 1 Department of Health Outcomes and Biomedical Informatics, University of Florida, 2199 Mowry Road, Gainesville, FL 32611, USA; rmkuu@ufl.edu (R.S.M.); hdcho5016@ufl.edu (H.D.C.); jenwoodard@ufl.edu (J.N.W.); bianjiang@ufl.edu (J.B.); eshenkman@ufl.edu (E.A.S.); yiguo@ufl.edu (Y.G.); 2 Cancer Informatics Shared Resource, University of Florida Health Cancer Center, 2033 Mowry Road, Gainesville, FL 32610, USA; 3 Community Outreach and Engagement, University of Florida Health Cancer Center, 2033 Mowry Road, Gainesville, FL 32610, USA; 4 Division of Hematology and Oncology, Department of Medicine, University of Florida, 1600 SW Archer Road, Gainesville, FL 32610, USA; fkaye@ufl.edu

**Keywords:** cancer, screening, cervical, colorectal, lung, breast, urban, poverty

## Abstract

**Simple Summary:**

Despite advances in cancer screening, late-stage cancer diagnosis is still a major cause of morbidity and mortality in the United States. In this study, we aim to understand demographic and geographic factors associated with receiving a late-stage diagnosis of lung, colorectal, breast, or cervical cancer, four cancers that have efficacious screening protocols. We find that in addition to the large body of research showing rurality is associated with proximal barriers to accessing screening, urban poverty was also significantly associated with being diagnosed with late-stage disease. Communities of urban poverty experience unique barriers to accessing cancer screening.

**Abstract:**

Despite advances in cancer screening, late-stage cancer diagnosis is still a major cause of morbidity and mortality in the United States. In this study, we aim to understand demographic and geographic factors associated with receiving a late-stage diagnosis (LSD) of lung, colorectal, breast, or cervical cancer. (1) Methods: We analyzed data of patients with a cancer diagnosis between 2016 and 2020 from the Florida Cancer Data System (FCDS), a statewide population-based registry. To investigate correlates of LSD, we estimated multi-variable logistic regression models for each cancer while controlling for age, sex, race, insurance, and census tract rurality and poverty. (2) Results: Patients from high-poverty rural areas had higher odds for LSD of lung (OR = 1.23, 95% CI (1.10, 1.37)) and breast cancer (OR = 1.31, 95% CI (1.17,1.47)) than patients from low-poverty urban areas. Patients in high-poverty urban areas saw higher odds of LSD for lung (OR = 1.05 95% CI (1.00, 1.09)), breast (OR = 1.10, 95% CI (1.06, 1.14)), and cervical cancer (OR = 1.19, 95% CI (1.03, 1.37)). (3) Conclusions: Financial barriers contributing to decreased access to care likely drive LSD for cancer in rural and urban communities of Florida.

## 1. Introduction

Globally, there are over 19.3 million cases of cancer reported and over 10.0 million cancer deaths annually [1]. Cancer is the second-leading cause of mortality in the United States (US), with ~609,360 deaths attributed to cancer in the year 2022 [2]. Screening programs can identify the risk for some cancers at an early stage and prevent severe morbidity, burdensome treatment, and mortality. Increasing cancer screening rates, a key public health priority of Healthy People 2030, will ensure earlier diagnosis of cancer and lead to higher survival rates [3]. Early detection leads to better prognosis through increased opportunities for successful treatment [4]. Timely screening can improve the efficacy of any cancer treatment. For colorectal and cervical cancer, early screening can lead to the avoidance of the cancer altogether through the identification and removal of abnormal cells and precancerous lesions [5,6]. Most notably, cervical cancer can potentially be eliminated from the US population through vaccination, screening, and early treatment [7]. While primary prevention is always the best treatment, secondary prevention via timely screening to detect cancers at early stages would greatly reduce current disparities in survival rates among demographic groups [8].

The early-stage diagnosis of cancer or pre-cancer is a key public health priority that will reduce the disease and financial burden to patients and their families [9]. Despite cancer screening being associated with significantly better cancer survival, many screening-eligible individuals do not receive cancer screenings recommended by evidence-based guidelines, leading to higher rates of late-stage cancer diagnosis in some populations [10,11]. Screening disparities are persistent among the same populations who experience higher cancer mortality, i.e., socioeconomically disadvantaged groups, especially those without health insurance, leading to higher rates of late-stage cancer diagnosis [11]. Rural–urban disparities in cancer mortality have long been acknowledged, and these disparities are associated with poverty and tobacco use in rural areas [12,13]. The survival of rural patients has long trailed the survival of urban patients [12], and patient travel distance has been suggested as a barrier to healthcare in rural areas [14,15]. In rural areas, screening disparities may also be associated with both lower insurance rates and the lower availability of healthcare resources [15,16].

Lung cancer is the most common cancer in the US and has the greatest cancer mortality, making up a quarter of all US deaths by cancer [17]. In 2022, the US Preventive Services Task Force (USPSTF) recommended yearly lung cancer screening with Low-Dose Computed Tomography (LDCT) for adults 50 to 80 years who have a 20-pack-year smoking history (at least a pack each day for 20 years) and currently smoke or have quit within the past 15 years [18]. Racial minority lung cancer patients face worse outcomes than non-Hispanic White patients, being less likely to receive recommended treatment and thus having a higher likelihood of cancer being diagnosed at a later stage [19]. Awareness of lung cancer screening is lower than that for other cancers. Even in higher cancer-burdened populations, those eligible may not have discussed the modalities of screening and screening programs with their providers [20].

Colorectal cancer (CRC) is the second leading cause of cancer death in the US and the third leading worldwide [21]. Although CRC is highly preventable through early screening, high mortality rates persist [9]. The USPSTF recommends a colorectal cancer screening for adults 45 to 75 years and for clinicians to selectively offer screening for colorectal cancer in consideration of the patient’s overall health, prior screening history, and preferences for adults aged 76 to 85 years [18]. Only 65.7% of adults ages 50 to 75 years self-report having been screened [22]. The incidence of CRC is increasing in younger populations, and its prevalence within the population varies among racial and ethnic groups [23]. Barriers to screening include not having a companion to escort and transport the patient home from the procedure, fear of the procedure, limited funds to purchase preparation materials, and a lack of education that hinders the comprehension of preparation instructions [22].

Breast cancer is the leading cancer among women in the US and globally and the leading cause of cancer-related morbidity, mortality, and years of life lost [4]. The global burden of breast cancer varies greatly among countries and is rising fast in some economically transitioning countries due to increased coverage of cancer screening [24]. From 1975 to 1990, female breast cancer mortality rates in the US increased annually. For three decades since 1990, breast cancer mortality rates have fallen annually in the US due to increased mammography screening and improved treatment, averting hundreds of thousands of deaths [4]. The USPSTF recommended biennial screening mammography for women aged 50 to 74 years, although screening recommendations for breast cancer may be updated soon. The American Cancer Society (ACS) recommends annual mammograms starting at 45, given the increasing rates of incidence in younger ages [18]. Substantial advances have occurred in identifying early-stage breast cancers through increased screening, but only some populations are benefiting. Urban areas and those of higher income have a significantly higher incidence rate of early-stage breast cancer than rural or low-income areas [13]. Increases in screening mammography have only marginally reduced the rate at which women present with advanced cancer [25], and mortality from breast cancer is still higher for those with a low income [13]. Different population groups have failed to benefit from early screening, including the elderly, those with low income, those from rural areas, and those with less education [26].

Cervical cancer is no longer the leading cause of cancer death among women in the US because of cancer screening, which results in detecting cervical abnormalities before the development of the disease [27]. Globally, cervical cancer is the fourth most common cancer among women, but it could be eliminated through focused vaccination screening and treatment programs [28]. Cervical cytology alone, to detect cellular changes in cervical tissue, is recommended by USPSTF every 3 years for women aged 21–65 years. Primary HPV testing is recommended to detect the presence of HPV and includes genotyping to determine high-risk (hrHPV) vs. low-risk HPV strains. HPV testing, either alone or alongside cervical cytology (i.e., co-testing), is recommended every 5 years in women ages 30–65 years [29]. HPV testing is also recommended and preferred as the most sensitive test for cervical cancer by the ACS, starting at age 25, given the causal relationship between persistent hrHPV infection and cervical cancer [30]. HPV is the leading cause of cervical cancer, and HPV vaccination is the primary prevention mode for cervical cancer. HPV vaccination rates lag in rural counties, and rural counties have a higher incidence of cervical cancer than urban counties [16,31]. The majority of women who are diagnosed with cervical cancer have never been screened before [32], meaning they lack the benefit of preventative healthcare services. Characteristics of women who receive late-stage cervical cancer diagnosis include those who do not have insurance or Medicaid, those who live in a low-income or rural area, and those who belong to an underserved racial/ethnic group [16].

In this study, we aimed to understand sociodemographic and geographic factors associated with receiving a late-stage diagnosis (LSD) of lung, colorectal, breast, or cervical cancer in the state of Florida. In addition to considering the contributions of race, age, gender, and insurance coverage toward receiving an LSD when a screening modality was available, we also considered the location of a patient’s residence. In doing so, we aimed to better inform future precision care interventions for demographic and geographic populations suffering from disparities.

## 2. Materials and Methods

We obtained demographic and tumor data for patients residing in Florida diagnosed with lung, breast, colorectal, or cervical cancer between 2016 and 2020 from the Florida Cancer Data System (FCDS), a statewide population-based cancer registry supported by the Florida Department of Health [33]. Out of 227,340 Florida residents who were diagnosed with lung, colorectal, breast, or cervical cancer, 21,607 patient records (9.5%) were excluded from the model due to missing staging information (7.0%), residential census tract (1.8%), valid age (0.1%), binary gender (0.03%), or race or ethnicity (0.7%). Age was grouped as follows: 20–49, 50–64, 65–75, and >75 years. LSD was defined using the TNM Number staging system (both clinical and pathologic staging with priority on clinical staging) as 3 (regional) or 4 (distant), while early stage was 1 (in situ) or 2 (localized) [34]. Primary insurance type at diagnosis was categorized as Private, Medicare, Medicaid, No Insurance/Self-pay, Other Governmental, or Insurance NOS/Other insurance or Unknown. Rurality was defined using USDA’s census tract based rural–urban commuting area (RUCA) codes and the commonly assigned categorization of RUCA codes 1–3 as urban and RUCA 4–10 as rural [35]. An estimate for neighborhood-level poverty for the residential census (2018) was obtained from Census.gov [36].

**Figure 1:**
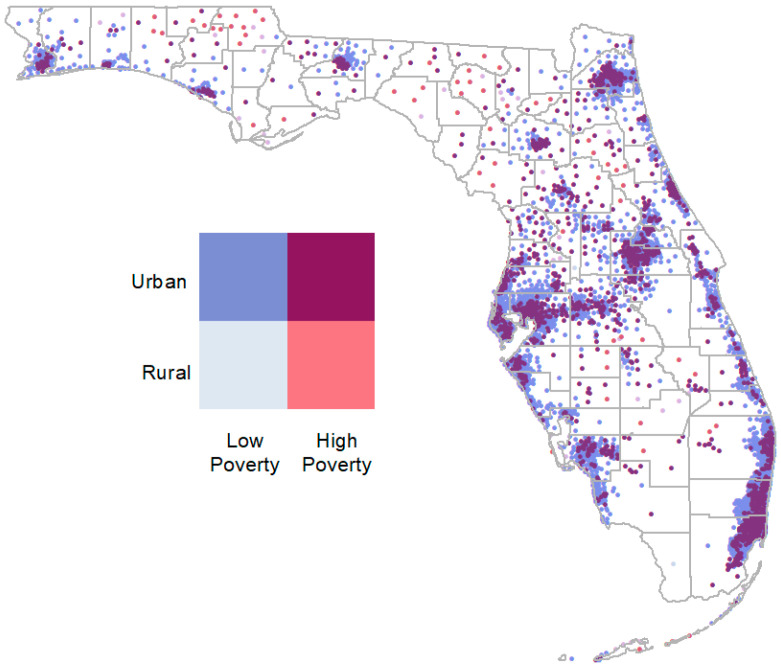
Map of centroid of census tracts to show general distribution or communities in Florida. Rurality and poverty combined into four categories. Urban poverty is shown in maroon.

Multi-variable logistic regression for each cancer type was used to analyze the odds of the health outcome of ‘having an LSD’ while controlling for age, sex, race, Hispanic ethnicity, insurance type, census tract rurality, and census tract poverty. Rurality and poverty were combined to make 4 categories: low poverty/urban, low poverty/rural, high poverty/urban, and high poverty/rural. Statistical analyses were performed using the SAS 9.4 (SAS Instutite Inc., Cary, NC, USA) logistic procedure with LSD as the binary outcome [37]. We tested for the multicollinearity of the categorical predictor variables using the linear regression procedure. The collinearity diagnostics showed no variance inflation on any predictor variable for each of the four cancers modeled. Figure 1 represents the distribution of census tracts in Florida by showing the centroid of each residential tract. Urban/high-poverty census tracts are purple/berry in color and tend to be clustered near larger urban areas.

## 3. Results

### 3.1. Summary Statistics

Between 2016 and 2020, there were 84,175 cases of lung, 51,700 cases of colorectal, 86,224 cases of breast, and 5227 cases of cervical cancer in Florida. Non-Hispanic White patients accounted for 80.0% of lung cancers but only 54.7% of cervical cancers (Table 1). Non-Hispanic Black patients accounted for 7.9% of lung cancer patients and 18.1% of cervical cancer patients. Hispanic patients accounted for 9.8% of lung cancer patients and 23.1% of cervical cancer patients. The percent of cervical cancer patients with Medicaid or no insurance was 25.4%, while only <10% of patients with lung, colorectal, or breast cancers were underinsured. Almost half of all cervical cancer patients resided in high-poverty urban communities, while only about a third of patient populations for other cancers resided in areas of urban poverty. In contrast, sixty-four percent of breast cancer patients were from low-poverty urban areas.

The percent LSD for each cancer was as follows: lung was 65.3%, colorectal was 57.7%, breast was 32.6%, and cervical was 51.1% (Table 2). The percent LSD for each cancer for patients aged 22–49 cancer were as follows: lung was 77.7%, colorectal was 67.2%, breast was 45.7%, and cervical was 43.2%. Cervical cancer is unique in that it impacts individuals aged 20–49 more than those over age 75. For each cancer, uninsured patients experienced a higher percentage of LSD. Low-poverty rural patients have more LSD than low-poverty urban patients, especially for cervical cancer, where low-poverty rural patients have 56.9% LSD compared to 50.3% for low-poverty urban patients. Before controlling for confounding factors, the percentage of patients diagnosed with late-stage cancer in high-poverty rural and high-poverty urban communities is similar.

### 3.2. Multi-Variable Results

Multi-variable logistic regression analysis results examining the odds of LSD while controlling for sociodemographic characteristics are presented in Table 3. Cervical is the only cancer where older patients have higher odds for an LSD, and those over 75 years of age have three times the odds of an LSD than those under 50 years (OR = 2.94 95% CI (2.94–3.98)). Moreover, those over 65 diagnosed with lung, colorectal, and breast cancer are less likely to have experienced an LSD. In lung cancer patients, men were 33% more likely than women to have an LSD (OR = 1.33 95% CI (1.29–1.38)). Non-Hispanic White patients with cervical cancer had no significantly different risk of having an LSD than other racial or ethnic groups, while non-Hispanic White patients had less risk of developing an LSD than non-Hispanic Black patients with lung, colorectal, or breast cancer patients. Non-Hispanic Black colorectal cancer patients had 14% higher odds of an LSD than non-Hispanic White patients (OR = 1.14 95% CI (1.07, 1.21)). Non-Hispanic Black breast cancer patients had 57% higher odds of an LSD than non-Hispanic White patients (OR = 1.57 95% CI (1.50, 1.65)). Lung cancer patients who were non-Hispanic Black (OR = 1.12 95% CI (1.05, 1.20)) or non-Hispanic other (OR = 1.18 95% CI (1.04, 1.34)) had higher odds of having an LSD than non-Hispanic White patients. Patients of all cancers who were uninsured or insured by Medicaid had higher odds of having an LSD than those with private insurance. Colorectal cancer patients who were privately insured had less chance of an LSD than those of all other insurance types.

Compared to those from low-poverty urban areas, patients from high-poverty urban areas saw higher odds of an LSD for lung (OR = 1.08 95% CI (1.04, 1.12)), breast (OR = 1.14, 95% CI (1.10, 1.18)), and cervical cancer (OR = 1.16, 95% CI (1.02, 1.32)). Patients from high-poverty rural areas had higher odds of an LSD for lung (OR = 1.23 95% CI (1.10, 1.37)) and breast cancer (OR = 1.32 95% CI (1.17, 1.47)). Even women from low-poverty rural areas saw higher odds of an LSD of breast cancer (OR = 1.24, 95% CI (1.07, 1.44)) than those from low-poverty urban areas. Patients from rural areas, whether high or low poverty, saw no greater odds of an LSD of cervical cancer. Only patients from high-poverty urban areas saw higher odds for LSD of cervical cancer. No rurality/poverty association was found for colorectal cancer, and the variable was excluded from the colorectal model.

## 4. Discussion

Similar to prior studies, we found significant associations between receiving a late-stage cancer diagnosis and sociodemographic factors, including race and ethnicity, age, insurance status, and rurality [37]. In our models, rural poverty greatly contributed to the odds of receiving an LSD for lung and breast cancer but not colorectal or cervical cancer. High-poverty urban census tracts demonstrated an additional burden related to LSD even when controlling for the well-known cancer risk factors and insurance coverage, especially for cervical cancer. Significantly greater odds of receiving an LSD were found for patients residing in urban poverty communities for lung, breast, and cervical cancer compared to urban communities of higher wealth. This demonstrates the importance of disaggregating large urban areas and properly differentiating the differing burdens related to social determinants of health for communities of higher and lower incomes within the same larger urban area. Studies have found rurality and patient travel distance may be a barrier to preventive healthcare [14,16]. This study did not find an additional burden related to rurality for patients from wealthier rural communities for lung, colorectal, or cervical cancer.

Poverty is a major cause of disparities in cancer health outcomes, arguably more influential than rurality [13]. Poverty, tobacco use, and a lack of insurance are burdens faced by both rural and urban communities [38,39]. Preventive health visits, such as cancer screenings, are underutilized in inner-city populations [22] and in neighborhoods with low education and low income [26]. There is a need for practical solutions that increase access to screening services and reduce barriers to services in low-income communities [16,40]. Adults who live alone with a lack of social contact have been shown to lack preventive healthcare services [41]. Older adults living in areas of poverty that also suffer from community violence report this lack of social support [42]. In urban areas with community violence, social withdrawal can be seen as a survival strategy while simultaneously leading to poor health outcomes due to the underuse of available medical services [42].

Cancer is a disease of old age, where mutations accumulate in our tissues throughout life; some of the mutations may contribute to cancers [43]. However, cancer patients under 50 are more likely to face an LSD for lung, colorectal, and breast cancer. As we have found, late-stage disease is associated with a young age at presentation in studies of many cancers, such as lung, colorectal, and breast cancer [44,45,46]. Early-onset cancer is often biologically different than later-onset cancers and can have a more aggressive presentation and clinical course, making early detection more crucial. Cervical cancer follows a different trend, with the odds of LSD of cervical cancer being greater among older women, especially those over 75 years of age. Cervical cancer screening guidelines through 2023 only recommend cervical and HPV screening for women up to age 65 [18]. Meanwhile, almost half of cervical cancers in older women are diagnosed at a late stage. These older women receiving LSD represent vulnerable patients who had not been receiving consistent screenings or appropriate follow-up at younger ages [47]. Other recent studies have supported the need to update the guidelines for cervical cancer screening for women over 65 years of age [47].

Insurance coverage is one of many challenges facing underserved populations [48]. A lack of health insurance limits access to care and can lead to health disparities. States that expanded Medicaid insurance coverage with the Patient Protection and Affordable Care Act (ACA) in 2011 saw an increase in cancer screenings for vulnerable groups and early-stage cancer diagnoses [49,50] and a significant decline in the proportion of newly diagnosed cancer patients who were uninsured [51]. Those states that did not expand Medicaid saw lower screening rates and widening screening disparities related to race and socioeconomic status due to larger numbers of uninsured [52]. However, patients with Medicaid continue to face persistent barriers to care due to low reimbursement rates, yet when providers are better incentivized to serve Medicaid patients, receipt of preventative cancer screenings increases [15]. The ACA has increased access to diagnostic services and treatment with the dependent coverage expansion provision, allowing young adults to be covered under their parents’ health insurance until age 26 [53]. The ACA dependent care expansion has been shown to increase the identification of early-stage cervical cancer in young women 21 to 26 years of age, potentially preserving the fertility capabilities of hundreds of young women [54].

Our findings indicating patients with Medicaid were more likely to be diagnosed with an LSD may also be related to screenings through the CDC’s Breast and Cervical Cancer Early Detection Program, which provides free screening for women who are uninsured [55]. The program served 13,022 clients and provided 22,369 screenings and diagnostic services in the 2019–2020 fiscal year [56]. Once diagnosed with breast or cervical cancer, eligible women are enrolled in Medicaid for treatment, thus possibly explaining our findings of a higher representation of women with Medicaid with LSD cervical cancer.

Interventions must expand quality health insurance coverage [25] and must also address other difficulties experienced by vulnerable populations in accessing care [11]. Breast cancer screening interventions intended to reduce the percentage of women who are diagnosed with late-stage disease have successfully increased breast cancer screening rates (before the disruptions of 2020) [57]. Still, ~28,000 late-stage breast cancers occurred in Florida during the study period (2016–2020), and our investigation reveals strong disparities in LSD of breast cancer, indicating early screening implementation was not successful for some sociodemographic groups.

Reported barriers to screening include poor awareness, fear of the procedure, limited funds to purchase preparation materials, inability to read or comprehend preparation instructions, hardship in being contacted or scheduling appointments, and not having a companion to escort and transport the patient home from the procedure [22,58]. These are challenges that affect minority and low-income communities, both rural and urban. Along with difficulty receiving time off work or help with childcare commitments [59], patients also may cite their fear of a diagnosis and lack of guidance with management of a positive cancer result as a barrier [60].

Cancer incidence and cancer mortality are known to be higher in high-poverty communities, regardless of rurality [13]. Communities of urban poverty, which may be geographically close to large healthcare systems, continue to experience persistent barriers to achieving health equity, including preventative cancer screening. Poverty has strong implications for health behaviors and health outcomes [61]. All communities of poverty suffer from economic vulnerability arising from various challenges, such as precarious employment and under-employment, poor access to quality public goods and services, and environmental and health risks. Moreover, urban communities of poverty are also burdened with socio-spatial segregation, violence, and insecurity [62]. Residential racial segregation and poor health remain critical issues impacting urban communities and represent historically rooted processes that continue to contribute to health inequalities [63].

We recognize a major limitation of this study is the absence of patient-level self-reported cigarette smoking history. High smoking rates account for a large portion of the cancer health disparity in rural areas. Some research has shown that measures of neighborhood-level poverty serve as stronger predictors of lung cancer risk even when controlling for smoking [64]. However, like most state cancer data systems in the US at this time, after much internal discussion with quality control and in consultation with the CDC, the FCDS has suspended the use of the Tobacco Use fields. These fields have had less reliability recently due to the rise of alternative nicotine delivery modalities. We also recognize that we do not have data on actual screening behavior but are using the stage at diagnosis to reflect early or late screening. In addition, we do not have individual income information and, thus, have extrapolated neighborhood poverty from the patient’s residential census tract. Income-related information is rarely included in large health datasets, leaving researchers to rely on spatial linkages to the census tract ‘neighborhood’ to acquire related socioeconomic indicators and infer socioeconomic status.

## 5. Conclusions

This study reveals how low-income urban areas, in addition to rural areas, represent unique challenges to achieving the goals of decreasing disparities in cancer health outcomes. Greater insurance coverage is not the only needed intervention to decrease disparity in LSD. The risk of being diagnosed at a later stage and incurring increased morbidity and mortality from a cancer that has an efficacious screening modality is higher for residents of high-poverty urban communities, regardless of race or insurance status. Consideration of patient-reported barriers to screening can help inform the implementation of future screening interventions and national screening programs [20]. Patient navigation is a healthcare service delivery system with the goal of eliminating barriers to preventive care, providing timely diagnosis, and implementing the best-practice treatment of cancer [65]. Patient navigation and coordination have been associated with greater preventive care, such as increased screening rates and a decrease in racial disparities [66]. Clinical data can be used within a learning health system to identify specific patients who are due for, have never been scheduled for, or did not keep a previously scheduled cancer screening or follow-up appointment [22]. More research is needed on the cost-effectiveness of targeted interventions, which increase adherence to continued screening, follow-up after abnormal initial screening test results, and other intensive interventions.

## Figures and Tables

**Table 1:** Demographic breakdown of Florida cancer diagnoses, 2016–2020. Urban is census tract RUCA 1–3; rural is census tract RUCA 4–10.

	Lung	Colorectal	Breast	Cervical
	(*n* = 84,175)	(*n* = 51,700)	(*n* = 85,371)	(*n* = 5232)
Age				
20–49	1898 (2.3%)	5040 (9.7%)	13,230 (15.3%)	2296 (43.9%)
50–64	20,698 (24.6%)	15,510 (30.0%)	28,404 (32.9%)	1683 (32.2%)
65–75	32,913 (39.1%)	15,326 (29.6%)	27,791 (32.2%)	810 (15.5%)
>75	28,646 (34.0%)	15,725 (30.4%)	16,799 (19.5%)	438 (8.4%)
Sex				
Women	40,689 (48.3%)	24,106 (46.6%)	85,371 (100%)	5232 (100%)
Men	43,464 (51.6%)	27,579 (53.3%)		
Race–Ethnicity				
Non-Hispanic White	67,308 (80.0%)	35,814 (69.3%)	60,057 (69.6%)	2864 (54.7%)
Non-Hispanic Black	6621 (7.9%)	6094 (11.8%)	9886 (11.5%)	945 (18.1%)
Non-Hispanic Other	1422 (1.7%)	1252 (2.4%)	2337 (2.7%)	173 (3.3%)
Hispanic	8241 (9.8%)	8173 (15.8%)	13,299 (15.4%)	1209 (23.1%)
Payer				
Private	13,892 (16.5%)	14,870 (28.8%)	32,982 (38.2%)	2158 (41.2%)
No insurance	2321 (2.8%)	1983 (3.8%)	2240 (2.6%)	513 (9.8%)
Medicaid	4218 (5.0%)	2856 (5.5%)	3610 (4.2%)	818 (15.6%)
Medicare	53,979 (64.1%)	27,024 (52.3%)	40,551 (47.0%)	1172 (22.4%)
Other Governmental	2558 (3.0%)	1003 (1.9%)	1507 (1.7%)	83 (1.6%)
Unknown	7207 (8.6%)	3964 (7.7%)	5343 (6.2%)	488 (9.3%)
Poverty–Rurality				
Low poverty, urban	49,505 (58.8%)	30,401 (58.8%)	55,453 (64.3%)	2591 (49.5%)
Low poverty, rural	1078 (1.3%)	612 (1.2%)	848 (1.0%)	51 (1.0%)
High poverty, urban	29,896 (35.5%)	18,563 (35.9%)	26,982 (31.3%)	2405 (46.0%)
High poverty, rural	2161 (2.6%)	1154 (2.2%)	1420 (1.6%)	98 (1.9%)

**Table 2:** Percent of patients within each demographic group diagnosed with late-stage cancer.

	Lung	Colorectal	Breast	Cervical
	65.3	57.7	32.4	51.1
Age				
20–49	77.7	67.2	45.7	43.2
50–64	73.2	60.6	35.2	56.4
65–75	64.6	54.9	26	58.1
>75	59.6	54.6	27.6	59.4
Sex				
Women	63.5	57.2	32.4	51.1
Men	66.9	58.1		
Race–Ethnicity				
Non-Hispanic White	64.9	57.2	29.6	52.6
Non-Hispanic Black	69.4	59.9	43.4	53.3
Non-Hispanic Other	69.8	59.5	35.6	45.1
Hispanic	65.2	58.4	36.6	47.1
Payer				
Private	71.1	60.2	35.2	44.6
No insurance	81.3	68	51.6	61.8
Medicaid	79.3	67.1	51.3	57.3
Medicare	66.1	56.8	27.6	60.1
Other Governmental	61.6	59.5	33.0	44.6
Unknown	36.0	41.5	30.9	38.1
Poverty–Rurality				
Low poverty, urban	64.6	57.9	30.7	50.3
Low poverty, rural	66.0	58.5	34.6	56.9
High poverty, urban	66.6	57.8	35.9	52.5
High poverty, rural	66.4	56.3	36.7	52.0

**Table 3:** Multi-variable logistic regression for stage at diagnosis as outcome variable. Florida Cancer Data System data years 2016–2020. Confidence intervals were provided at *p* < 0.05. Red and blue color indicates significant odds assocated with increased, or decreased LSD, respectively.

	Lung	Colorectal	Breast	Cervical
	(*n* = 84,175)	(*n* = 51,700)	(*n* = 85,371)	(*n* = 5232)
Age				
20–49	Reference	Reference	Reference	Reference
50–64	0.82 (0.72, 0.94)	0.76 (0.71, 0.82)	0.67 (0.64, 0.70)	1.97 (1.71, 2.27)
65–75	0.59 (0.51, 0.67)	0.61 (0.57, 0.67)	0.45 (0.43, 0.48)	2.06 (1.64, 2.60)
>75	0.52 (0.46, 0.60)	0.69 (0.63, 0.75)	0.52 (0.49, 0.56)	2.94 (2.17, 3.98)
Sex				
Women	Reference	Reference		
Men	1.33 (1.29, 1.38)	1.05 (1.01, 1.09)		
Race–Ethnicity				
Non-Hispanic White	Reference	Reference	Reference	Reference
Non-Hispanic Black	1.12 (1.05, 1.20)	1.14 (1.07, 1.21)	1.57 (1.50, 1.65)	1.02 (0.85, 1.21)
Non-Hispanic Other	1.18 (1.04, 1.34)	1.06 (0.94, 1.20)	1.15 (1.05, 1.25)	0.88 (0.63, 1.23)
Hispanic	1.00 (0.95, 1.06)	1.08 (1.03, 1.15)	1.18 (1.13, 1.23)	0.78 (0.67, 0.91)
Payer				
Private	Reference	Reference	Reference	Reference
No insurance	1.92 (1.69, 2.18)	1.54 (1.38, 1.72)	1.98 (1.80, 2.16)	2.38 (1.91, 2.97)
Medicaid	1.64 (1.50, 1.81)	1.51 (1.37, 1.66)	1.83 (1.70, 1.97)	2.11 (1.76, 2.53)
Medicare	1.04 (0.98, 1.09)	1.09 (1.03, 1.16)	1.07 (1.02, 1.12)	1.50 (1.21, 1.87)
Other Governmental	0.76 (0.69, 0.84)	1.24 (1.07, 1.43)	0.97 (0.87, 1.09)	0.99 (0.63, 1.58)
Unknown	1.27 (1.16, 1.40)	1.17 (1.07, 1.28)	1.26 (1.18, 1.35)	1.17 (0.93, 1.48)
Poverty–Rurality				
Low poverty, urban	Reference		Reference	Reference
Low poverty, rural	1.16 (1.00, 1.35)		1.24 (1.07, 1.44)	1.10 (0.61, 2.00)
High poverty, urban	1.08 (1.04, 1.12)		1.14 (1.10, 1.18)	1.16 (1.02, 1.32)
High poverty, rural	1.23 (1.10, 1.37)		1.31 (1.17, 1.47)	1.03 (0.66, 1.62)

## Data Availability

This study used data from the Florida Cancer Data System of the Florida Department of Health. Any published findings and conclusions are those of the authors and do not necessarily represent the official position of the Florida Department of Health. Requests for data for research purposes can be made through instructions on this website: https://fcds.med.miami.edu/inc/datarequest.shtml (accessed on 1 June 2023).
